# Unsupervised machine learning identifies early stage breast cancer subclasses from aberrant glycogene expression and informs personalised drug treatment

**DOI:** 10.1007/s44371-026-01070-0

**Published:** 2026-11-14

**Authors:** Mia Venter, Kevin J. Naidoo

**Affiliations:** 1 Scientific Computing Research Unit Address, University of Cape Town, PD Hahn Building, Rondebosch, Cape Town, 7701 South Africa; 2 Department of Chemistry, University of Cape Town, PD Hahn Building, Rondebosch, Cape Town, 7701 South Africa

## Abstract

Unsupervised hierarchical clustering of early-stage breast cancer glycogene expression data using the Growing Hierarchical Self Organising Representation Map (GHSORM) algorithm identified seven subclasses. These glycogene expression clusters (GECs) overlap with known breast cancer biomarkers such as hormone receptor status and immune signatures. Feature discovery via Recursive Feature Elimination with Strikeout (RFES) identified a panel of 60 glycogenes that can predict GECs on an independent testing set with 90.48% accuracy. GEC-based subtyping of patients from the I-SPY2 clinical trial revealed an 86% pathologic complete response (pCR), demonstrating how glycogene expression in combination with custom machine learning algorithms could be an approach to molecular cancer diagnosis capable of informing personalised drug treatment.

**Supplementary Information:**

The online version contains supplementary material available at https://doi.org/10.1007/s44371-026-01070-0.

## Introduction

Gene-expression based cancer subtyping inherently suffers from the curse of dimensionality, where the sample size is several orders of magnitude smaller than the number of genes [1]. Artificial intelligence (AI) and machine learning (ML) algorithms are increasingly being used to extract genomic trends common to sample sets that lead to a clustering of clinically meaningful classes.

Advancements in machine learning methods have made breast cancer subtyping on the basis of gene expression a reliable approach [2]. Correspondingly, gene expression assays such as the PAM50 [3, 4] and MammaPrint [5, 6], are utilised in clinical settings as breast cancer subtyping diagnostics. Despite these advances, a biological description of patient prognosis and an understanding of the treatment regimen that responds to the underlying biochemical fundamentals driving tumour progression, remains unmet [7].

The ongoing I-SPY2 clinical trial was designed to address this need. Focusing on high-risk, locally advanced early-stage breast cancer in women over 18 years of age, the trial aims to accelerate the discovery of novel drug combinations to determine which breast cancer subtypes will benefit [8]. Response Predictive Subtypes (RPS-5) were developed by retroactively assessing pre-treatment biomarker signatures and grouping patients accordingly with the goal of maximising pathologic complete response (pCR) [9]. In addition to Receptor Subtypes, derived from MammaPrint classification and hormone receptor status, RPS-5 accounts for proliferation markers and the likelihood of responding to immunotherapy. To date the approach has resulted in a 7% pCR improvement. The pre-treatment molecular profiles that are extracted in this trial represent a valuable resource, as well as an assessment of the efficacy of subtyping based sample treatment relative to clinical outcome.

Glycosylation is a non-template driven, highly orchestrated process in which carbohydrate moieties are conjugated to proteins and lipids. In cancer this process is dysregulated and known to be a feature of tumourigenesis [10]. The glycoconjugates that present themselves on the surface of epithelial cells as signalling [11] and immune receptors [12], play various roles in tumorigenesis [13–16]. The glycogenes that catalyse these reactions are located in specific areas along the ER-Golgi complex, and their abundance and spatiotemporal location determine the nature of protein and lipid glycosylation [17]. Glycogene expression can classify different cancer types and cluster breast cancer samples into clinically relevant groups [18]. The pervasiveness of glycosylation in cellular functionality and its correlation to hormonal and developmental aberrations provides an opportunity for improved breast cancer subtyping.

The Growing Hierarchical Self-Organising Map (GHSORM) [19] was developed to identify hierarchical class-representative structures in datasets. Here we apply the method to breast cancer glycogene expression data as a means toward subclass discovery. The algorithm first performs dimensionality reduction using a Denoising Autoencoder that ensures representative learning from high-dimensional data. Following this, the representation layer is mapped onto a series of hierarchically structured Self-Organising Maps (SOMs), each layer offering finer-grained resolution of the clustering space than the preceding one. GHSORM outperforms more rudimentary clustering techniques such as k-means and hierarchical clustering, particularly in cases where the data is hierarchical [19].

A rational explanation of ML-derived subtype models is essential for informing patient care following clinical diagnoses [20]. To this end, unsupervised methods such as GHSORM are useful for uncovering underlying data structures that drive sample clustering. However, follow-up analytics are necessary to determine how specific genes contribute to this clustering and their role in disease-causing mechanisms, which ultimately informs prognosis and treatment. We combine our unsupervised clustering algorithm with an in-house feature discovery Random Forest [21] wrapper, Recursive Feature Elimination with Strikeout (RFES), to provide the rationale for clustering, as well as a minimal set of glycogenes that can define clusters with maximal accuracy. Given the important role of the immune system in the early stages of breast cancer, and the importance of glycosylation in immune modulation, we investigate the co-occurring glycogene and immune signatures in each cluster. Finally, we use our glycosylation-based subtyping schema to group patients from the I-SPY2 clinical trial and assess the impact on pathologic complete response (pCR).

## Results

### Glycogene expression-based breast cancer subtype discovery with GHSORM

**Fig. 1:**
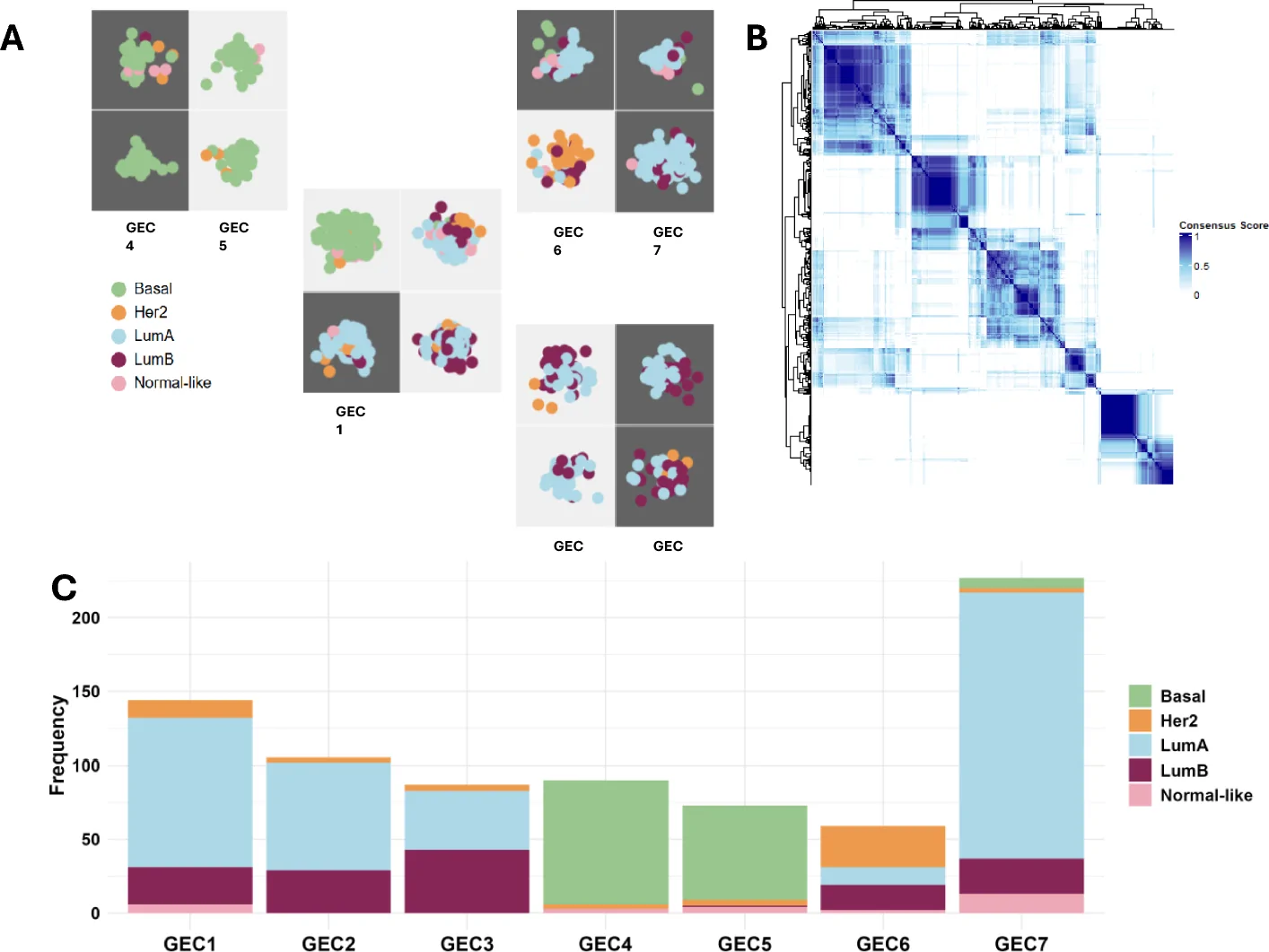
Unsupervised glycogene-based subtype discovery. **A** Glycogene Expression Clusters (GECs) identified by GSHORM. The parent map (middle) split into three daughter maps with 4 SOM nodes each. Samples (represented by dots coloured according to PAM50 subtype) that cluster close together share similar glycogene expression signatures. GECs are denoted by background colours and are labelled accordingly. **B** Consensus matrix showing sample to GEC stability during the hyperparameterisation process. **C** Proportion of PAM50 subtype in each GEC

Seven Glycogene Expression Clusters (GECs) were identified following the application of the unsupervised GHSORM subtype discovery algorithm to the TCGA data (Fig. 1A). The first SOM layer easily separates Basal from Luminal cancers, while the second layer provides a granular separation to uncover further clusters. Model hyperparameterisation was performed through a brute-force grid search approach that aimed to minimise the Davies-Bouldin Index (DBI), a measure of intra- vs inter-cluster similarity [22]. Clusters remained reasonably consistent throughout the hyperparamerisation process (Fig. 1B), eventually resulting in a DBI of 3.17.

The GECs overlapped quite extensively with PAM50 subtypes, and the Normalised Mutual Information (NMI) index [23] between PAM50 subtypes and GECs was 0.52 (Fig. 1C). GEC4 and GEC5 consisted mainly of Basal samples, while most of the Her2 samples were located in GEC6. The Luminal A and Luminal B (LumA, LumB) samples were spread throughout the remaining GECs, suggesting substantial molecular heterogeneity in these subtypes that extends beyond hormone receptor status. Neither the GEC nor the PAM50 subtyping schema resulted in prognostic significance (Supplementary Figure S1). This is likely due to exclusion of stage III and IV samples from the study. Excluding late-stage samples was necessary to evaluate the efficacy and value of glycosylation-based subtyping for informing early-stage breast cancer discovery and molecular phenotyping.

In comparison to this approach the application of commonly used static clustering techniques such as k-means and hierarchical clustering resulted in inferior model performance (DBI = 3.26 and 3.5 respectively) (Supplementary Figure S2).

### Recursive Feature Elimination with Strikeout (RFES)

A Recursive Feature Elimination with Strikeout (RFES) approach was developed to identify the glycogenes that significantly contribute to GEC classification. Here the RFES iteratively removed unimportant genes from a Random Forest classification model and terminated when model accuracy decreased be a set *strikeout* parameter (*s*). The introduction of the *strikeout* parameter allows the accuracy to fluctuate, thereby increasing the flexibility of the model and facilitating a more exhaustive search of the feature space.

**Fig. 2:**
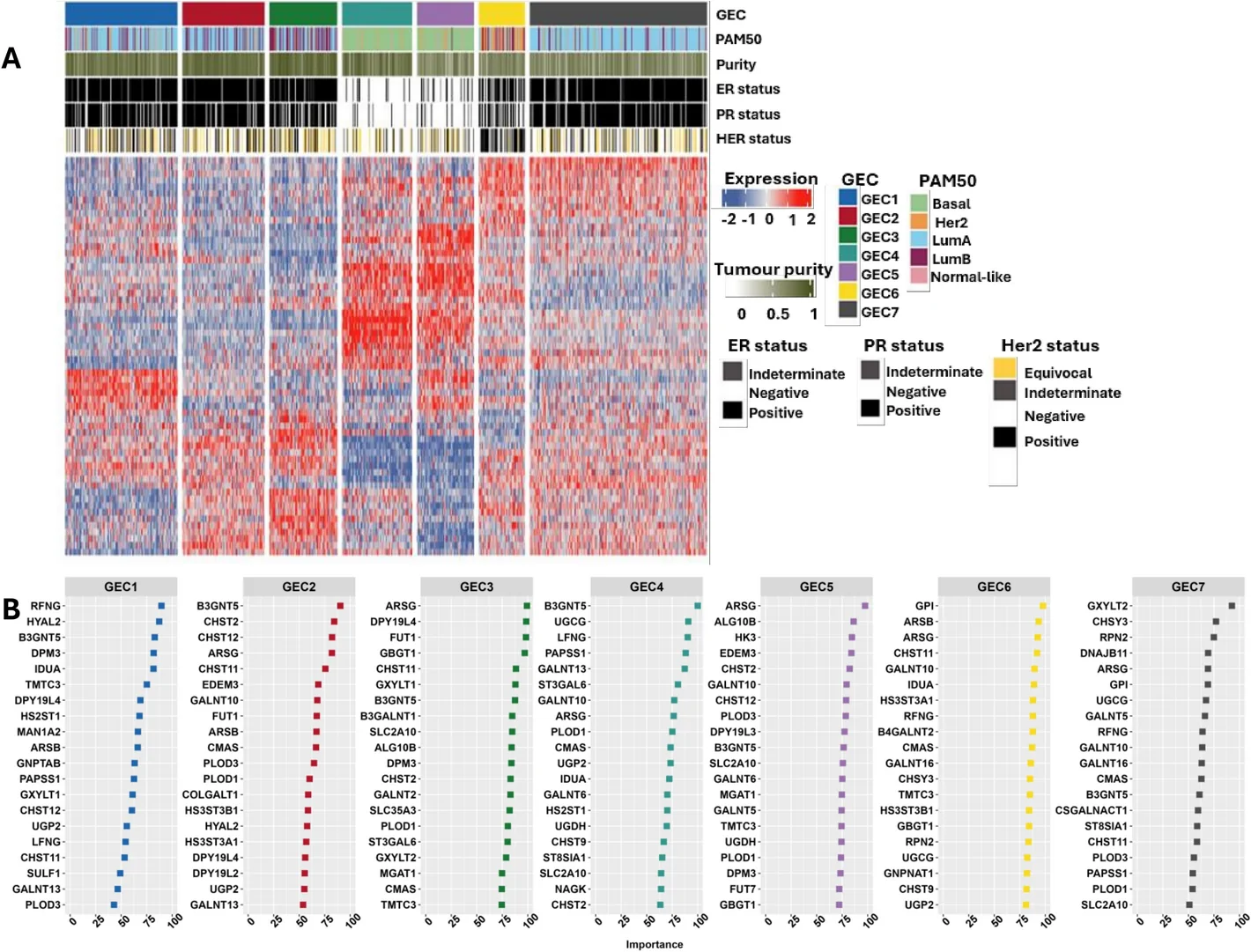
Supervised feature discovery with RFES.** A** Expression of the 60 glycogenes from the best-performing RFES model**.** ER = Estrogen Receptor, PR = Progesterone Receptor. **B** Variable importance for the top 20 most important genes in each GEC. The list of 60 glycogenes, including full gene names and RFES importance values, can be found in Supplementary Table S1

Using RFES, 60 glycogenes were identified that could classify samples into GECs within an accuracy of 90.48% using a 5-fold cross-validation approach on an independent testing dataset (Supplementary Table S1). Defined as a subset of the 341 total GEGs, the glycosignature is unique to each of the seven GECs (Fig. 2A). As a result, the glycogenes required to classify each GEC differ distinctly (Fig. 2B; Supplementary Figure S3). Model performance remains consistent between the seven classes (Supplementary Figure S4).

### GEC immune cell composition analysis

**Fig. 3:**
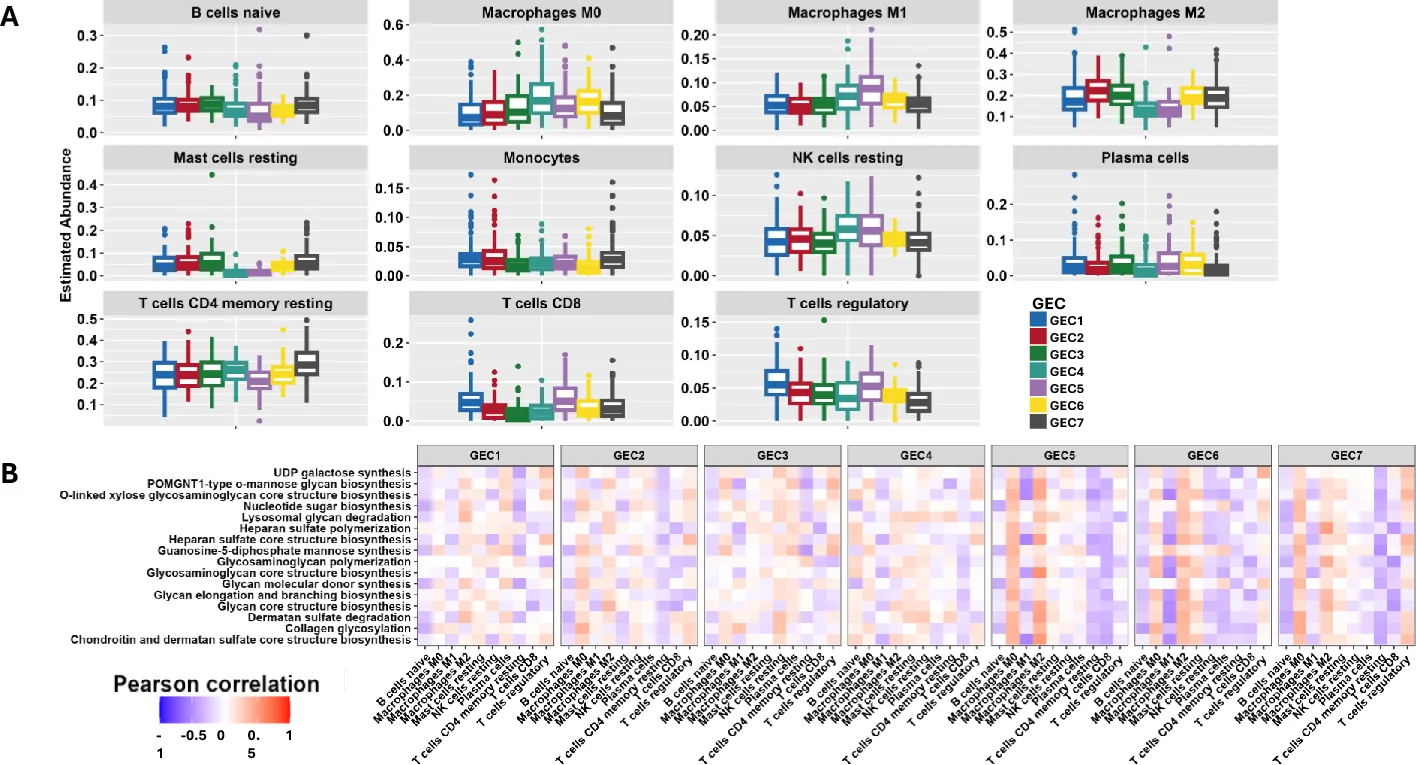
Immune landscape of the GECs.** A** Boxplots showing immune cell abundance for each GEC. Only significant immune cells are shown (pairwise Kruskal Wallis adjusted p-value < 0.05 and total cell abundance across samples > median cell abundance). **B** Pearson correlation coefficients between glycosylation pathways and estimated immune cell abundance for significant immune cell types as identified in **(A)**. Only significant cell – path combinations are included (correlation coefficient > 0.25 and adjusted p-value < 0.05; mean correlation coefficient across GECs > 0.25)

Undertaking immune cell deconvolution with Cibersort revealed significant variation of immune cell abundance between GECs (Fig. 3A; Supplementary Table S2). As an illustration despite similar PAM50 subtype distributions, GEC2 has an elevated abundance of Monocytes and CD8 T-cells in comparison with that of GEC3 (pairwise Wilcoxon adjusted p-value = 1 × 10^–3^ and 1 × 10^–2^ respectively). Similarly, there are clear differences between the two Basal clusters, GEC4 and GEC5, in T-cell abundance, where GEC5 has a greater amount of CD8 and regulatory T-cells (pairwise Wilcoxon adjusted p-value = 5.51 × 10^–8^ and 4.31 × 10^–6^, respectively), while GEC4 has a greater abundance of CD4 memory resting T-cells (pairwise Wilcoxon adjusted p-value = 4 × 10^–3^). Further tumour purity was strongly associated with GEC (Wilcoxon p-value < 2 × 10^–16^) (Supplementary Figure S5), potentially due to differences in glycogene expression profiles between different cell types in the tumour microenvironment.

The strong regulation of immune cells by glycosylation likely explains why glycogene expression can identify breast cancer subtypes with varying immune cell abundances and tumour purity. To explore this, we used Pearson correlation to investigate the relationship between glycosylation pathways and relative immune cell abundance (Fig. 3B). The patterns of covariance differed by GEC. A strong signal that emerged was the negative correlation of M1 macrophages and positive correlation of M2 macrophages with dermatan, chondroitin, and heparan sulphate biosynthesis, all glycosaminoglycans present in the extracellular matrix that play important roles in macrophage polarisation. This signal is particularly prevalent in GEC5-7 and is less pronounced in GEC1-4. In GEC3, activation of the guanosine − 5-diphosphate (GDP) mannose pathway is positively correlated with the abundance of regulatory T-cells and negatively correlated with CD4 memory resting T-cells (Supplementary Table S3).

### Clinical implications of glycogene-based subtyping

The heterogeneity observed in breast cancer is indicative of the variety in the mechanisms underpinning tumourigenesis, one of which is aberrant glycosylation. The pre-expression profiles generated by the I-SPY2 trial to classify 984 patients into GECs with RFES were used to test whether differential glycogene expression-based subtyping has clinical potential.

The I-SPY2 GECs have a similar PAM50 profile compared to the TCGA GECs, although the NMI between PAM50 subtypes and GEC is lower (NMI_I-SPY2_ = 0.32 vs NMI_TCGA_ = 0.52). Further, the expression profile of the 60 glycogenes is comparable to the TCGA (Supplementary Figure S6).

**Fig. 4:**
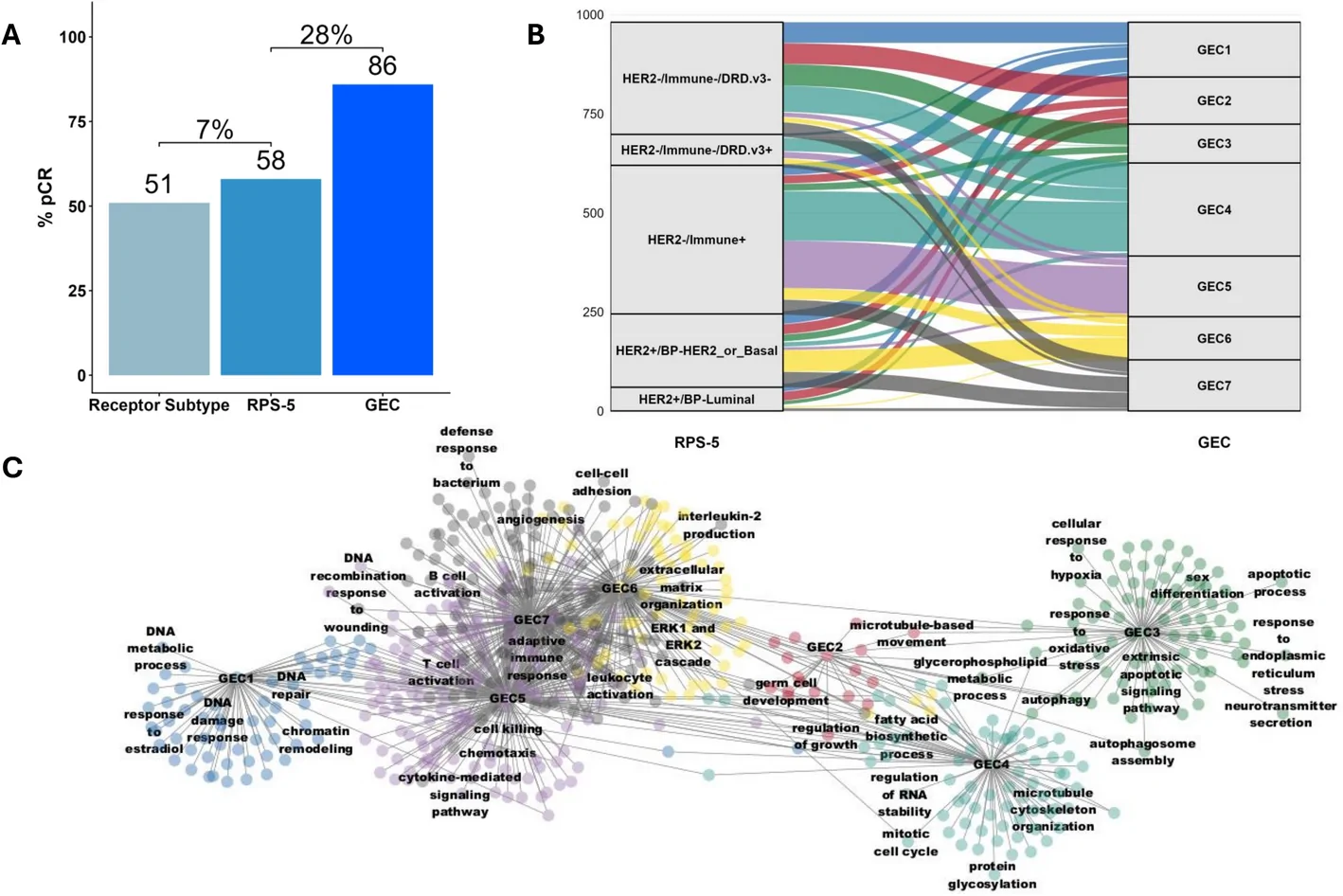
Characterisation of GECs in relation to clinical response and functional enrichment. **A** Estimated pCR rates if treatments had been assigned using Receptor Subtypes, RPS-5, or GEC. **B** Sankey diagram showing the relation of samples between RPS-5 and GEC. **C** Functional enrichment of the non-glycogenes predicted in each GEC. Nodes are coloured according to the GEC they were enriched in

In a previous study, the RPS-5 subtyping schema and retroactive grouping of the patients according to RPS-5 yielded a 7% improvement in pCR compared to Receptor Subtype [9]. Using the same method (detailed below in Materials and Methods), we estimated pCR had patients been grouped according to GEC (Fig. 4A, Table 1). Our glycogene-based subtypes resulted in 86% pCR, yielding a 28% improvement compared to the RPS-5 clustering procedure, and a 35% compared to Receptor Subtype. Surprisingly, the overlap between RPS-5 and GEC, and Receptor Subtype and GEC, is not outstanding (NMI = 0.12 and 0.17, respectively). This measure hides an important observation that was revealed through visual assessment of sample flow between RPS-5 and GEC (Fig. 4B). Notably there is a concordance between the Her2-/Immune subtype and GEC4 and GEC5, as well as between HER2+/BP-HER2_or_Basal and GEC6, which contains many Her2 + samples in the original TCGA data as well.

**Table 1:** Hyperparameters that led to optimal GHSORM performance

Hyperparameter	Value
Learning rate start	0.8
Learning rate end	0.02
Number of training steps for denoising autoencoder	100,000
T1	0.4
T2	0.05

All patients in the two Basal clusters, GEC4 and GEC5, that were receiving the optimal treatment (n = 3 and n = 9, respectively) achieved a pCR. The optimal treatment given was Paclitaxel + Pertuzumab + Trastuzumab in GEC4 and Paclitaxel + Pembrolizumab in GEC5. This affirms the existence of two Basal-like subtypes, that are each responsive to drugs regimes that target different underlying tumorigenesis mechanisms. The optimal treatment in GEC6 was Paclitaxel + AMG 386 + Trastuzumab, with 4 / 5 GEC6 patients in this treatment regime achieving pCR. The success of this treatment suggests that GEC6 samples are composed of a Her2-like, pro-angiogenic phenotype.

The 60-glycogene panel successfully groups patients into treatment-responsive subtypes. This suggests that the differential glycogene expression linked to glycan/glycoconjugate signalling which regulates cellular communication and tumorigenic pathway activation is unique to each GEC. To unpack this, a gene regulatory network was constructed where regression modelling was used to predict the expression of non-glycogenes using the 60 glycogenes as predictors (see Materials and Methods).

A functional profiling of the non-glycogenes was conducted to reveal potential associations for glycosylation in each GEC (Fig. 4C). Glycogene expression in each GEC associates with specific functions that influence treatment outcomes. In GEC5 (the immunotherapy-responsive Basal subtype), glycogene expression predicted genes driving immune function, including B- and T-cell activation. In GEC6, it predicted genes regulating angiogenesis, extracellular matrix organisation, and the ERK1/2 cascade. This finding provides a rationale for glycogene-based subtyping that may, with further research, lead to novel biomarkers and treatment targets that have mechanistic links of use for therapeutic design.

## Discussion

It is well known that Luminal and Basal cancers are genetically distinct [24]. Here the aberrant glycogene signatures foundational to each of these clinical subclasses produces separation in the first layer SOM (Fig. 1A). While this finding alone is not impressive, GHSORM's subsequent fine-grained disentanglement of the underlying genomic network, using differential glycogene expression data from a relatively small sample, produced two additional daughter maps and a total of seven GECs. The overlap between GEC and PAM50 subtype (Fig. 1C) has been previously reported by our group [18], and is indicative of the role that glycogenes play in the biology related to the PAM50 genes [4], including hormone [25–27] and growth factor receptor modulation [28]. However, the GECs moreover capture molecular signatures that are not captured by the PAM50 subtypes, as can be seen by the spread of LumA and LumB samples across GEC1, GEC2, and GEC7.

We developed RFES to identify the molecular signatures associated with each GEC, as well as to derive a minimally sized glycogene panel that could classify new samples according to GEC. Key considerations for RFES performance were to (i) maximise the model accuracy on an independent testing dataset, (ii) minimise the per-class classification error, and (iii) reduce to a minimum the number of genes in the final model. This approach resulted in the identification of a set of 60 glycogenes that could classify GECs with and accuracy of 90.48% (Fig. 2A, Supplementary Figure S6), outperforming other commonly used [29] Random Forest feature discovery algorithms (Supplementary Table S4). It is noted that the 60-glycogene panel does not have the necessary accuracy required for clinical use. Nevertheless, this provides promising insight into the approach required for differential glycogene-based biomarker discovery. Compared to other biomarkers discussed in the literature, its clinical value lies in the ability of the glycogene expression profile to precisely distinguish the distinct glycosylation linkages and tumourigenic drivers that make each subclass unique and therapeutically accessible (Fig. 2B, Supplementary Figure S3).

To illustrate this, consider that a number of the most important glycogenes in GEC6 are related to the sulphation of glycosaminoglycans (GAGs). ARSB and ARSG are sulfatases that hydrolyse sulphate groups on chondroitin and dermatan sulphate, while CHST11 catalyses the sulphation of chondroitin. Sulphated GAGs can create specific tumour microenvironments by building size-selective barriers around cells, thereby controlling what modulators cancer cells interact with. Furthermore, sulphated GAGs are highly sensitive signalling molecules, facilitating intercellular communication and acting as coreceptors that trigger activation of downstream signalling pathways that modulate cancer growth [30]. It is therefore not surprising that sulphated GAGs have diagnostic potential in several cancer types [31]. The importance of distinct glycogenes in each GEC likely points to diverse roles for glycosylation in tumorigenesis that mirror the heterogeneity of breast cancer. This may explain why the GECs capture molecular variation not identified by the PAM50 subtyping procedure.

An important objective in this study was to investigate the immune phenotypes associated with each GEC. This is of particular interest given the integral relationship between glycosylation and the immune system [32]. The strongest signal we observed was a positive correlation of M2 macrophage abundance, and a negative correlation of M1 macrophages, with the biosynthesis of sulphated GAGs, which was especially pronounced in GEC5 – 7. Macrophage polarisation is a process whereby tumoricidal M1 macrophages are re-educated by the tumour microenvironment to become immune dampening and pro-tumorigenic [33]. The polarisation of macrophages drastically alters the expression of GAG sulfotransferases, in turn influencing tumour cell proliferation [34]. The observation that GDP-mannose biosynthesis is positively correlated with the abundance of regulatory T-cells in GEC3 is interesting, since mannose may stimulate regulatory T-cell differentiation, thereby dampening immune responses [35]. The covariance between glycosylation pathways and immune cell abundance varies distinctly across GECs, indicating a context-dependent interplay between tumour immunity and glycosylation. These distinct profiles highlight new avenues for personalised treatment strategies aimed at specific immune-glycosylation axes.

Using a RFES model trained on TCGA data, I-SPY2 clinical trial patients were classified into GECs based on their pre-treatment 60-glycogene expression profiles. The pCR and treatment arm were mapped to GEC and RPS-5, following which the pCR outcomes based on GEC grouping performance was evaluated (Table 1). Notably, this subtyping approach yielded an estimated maximal pCR of 86%—a 28% improvement over the RPS-5 schema (Fig. 4A).

Although the number of patients receiving the same treatment in each GEC was small, the results are encouraging. In GEC5, all 9 patients who received Paclitaxel + Pembrolizumab achieved pCR. Pembrolizumab is a humanised anti-PD1 (Protein Death Ligand 1) antibody that alleviates the inhibition of T-cell responses in cancer. Tumour cell expression of PDL1 (Protein Death Ligand 1) may be an indicator of positive response to Pembrolizumab therapy [36]. Indeed, “immune-hot” hepatocellular carcinoma is more likely to respond to Pembrolizumab therapy [37], suggesting that a high degree of immune infiltration is important for a successful response. Gene Ontology profiling of our gene regulatory network shows that glycogene expression is associated with an immune response in GEC5 (Fig. 4C). This finding provides preliminary evidence that glycogene expression is integrated with the aspects of tumoral biology that can predict treatment response.

Similarly, the best treatment for GEC6 was Paclitaxel + AMG 386 + Trastuzumab. Trastuzumab is used for the treatment of Her2 + cancers, and binds the Her2 receptor, preventing its activation and causing tumour cell lysis [38]. In the absence of treatment, the activation of Her2 results in activation of downstream signalling pathways, including mTOR, Ras, and the Erk1 / Erk2 cascade [39]. AMG 386 is an angiopoietin antagonist that inhibits angiogenesis by preventing the binding between Ang1/Ang2 and Tie2 [40]. Glycogenes important for GEC6 classification can predict the expression of genes involved in the Erk1 / Erk2 cascade, as well as angiogenesis (Fig. 4C). The molecular signatures captured by the 60-glycogene panel appear to be closely related to treatment targets, providing a rationale for improved pCR if patients were to be grouped according to GEC.

This study has limitations that must be acknowledged. The relationship between glycogene expression and the extent of glycosylation is not established and a matter of debate as has been described by us recently [17]. First, the glycomic states of the GECs are not discussed here. Second, because the samples used in this study are derived from bulk tumour tissue, the specific cell types contributing to the observed glycosylation signatures remain unknown. As this work is a proof-of-concept study, functional validation experiments linking glycosylation to known tumourigenic pathways are required to fully realise the translational value of these findings. Moreover, incorporating glycogene expression into pre-existing subtyping schemas, such as RPS-5, may result in the further refinement of breast cancer subtypes.

Glycogene-based subtype discovery captures molecular signatures linked to fundamental aspects of tumour biology, from hormone receptor status to the immune microenvironment. This study demonstrates that applying specialised machine learning algorithms to aberrant glycogene expression profiles successfully identifies distinct cancer subtypes. Ultimately, this approach paves the way for discovering predictive biomarkers directly tied to treatment response, offering a robust framework for personalised cancer care.

## Materials and methods

### Data preparation

The TCGAbiolinks package [41] in R-4.4.1 [42] was used to download 1067 primary, untreated breast cancer and 111 matched normal RNA-seq samples from female patients from The Cancer Genome Atlas (https://www.cancer.gov/tcga). To ensure the applicability of our method to the stratification of early breast tumours, only 785 Stage I and II samples were used in this analysis (n_total_ = 785; n_LumA_ = 406; n_LumB_ = 139; n_Her2_ = 57; n_Basal_ = 155; n_Normal-like_ = 28).

Ensemble IDs were converted to HGNC symbols using the org.Hs.eg.db library [43], and in cases where duplicate symbols exist, the entry with the highest mean expression samples was used. Glycogenes and pathways were obtained from GlyocoEnzOnto [44]. Tidyverse [45] and data.Table [46] were instrumental in performing dataset manipulation.

Differential expression analysis was conducted using the DESeq2 [47] package and Log Fold changes were obtained using the “apeglm” method [48]. The variance stabilised data (using only the intercept in the design matrix) was used for further analysis.

### The Growing Hierarchical Self-Organising Map

We developed the Growing Hierarchical Self-Organising Representation Map (GHSORM) specifically for the purpose of cancer subtype discovery [19]. GHSORM relies on two components: the Denoising Autoencoder (DA) and the Self-Organising Map (SOM). These components can be combined in different ways to create several algorithmically similar models. To find the optimal algorithmic structure and model hyperparameters, a brute-force grid search approach was employed with the objective of minimising the Davies-Bouldin Index (DBI) between clusters. The best-performing model was a Pre-Processor GHSORM in which one DA is shared between all SOM layers. All 341 glycogenes with a standard deviation (SD) > 0.5 across samples was used as input to GHSORM.

At the encoder end, glycogene expression input features were corrupted by addition of Gaussian noise with a standard deviation of 0.2. The contribution of the corrupted input feature to the hidden layer was neuron and is defined by weights, *W* and biasing parameters, *b*. A hyperbolic tangent function, *f*, is applied to these that give the activation of the hidden neurons.

1 $$ y = f\left( {W\hat{x} + b} \right) $$

At the decoder end (the output layer) the samples’ reconstructed features, $\hat{x}$, are generated. This is done via a set of parameters biasing the neurons, $\hat{b}$, in the network’s layers accompanying the weights, $\hat{W}$. A hyperbolic tangent function, *f*, is once again applied to regulate of the contribution of the representation layer, *y*.

2 $$ \hat{x} = f\left( {\hat{W} y + \hat{b}} \right) $$

This procedure of learning features from artificially corrupted input data enables dimensionality reduction while still preserving the structure of the input data space.

The hidden representation layer is then mapped onto a SOM. Training the SOMnvolves an ordering and a convergence phase, which were implemented using one and ten epochs of samples respectively, according to the following cost function:

3 $$ K\left( {W, b, U} \right) = \mathop {\min }\limits_{d} \mathop \sum \limits_{e = 1}^{E} h_{de} \left\| {y - y_{e} } \right\|^{2} $$

where *E* is the total number of SOM neurons and *u*_*e*_ represents their codebook vectors. The neighbourhood function, *h*_*de*_, controls the surface elasticity of the neuron lattice.

Self-Organising Map growth and depth in the GHSORM algorithm are controlled by the user-provided parameters τ_1_ and τ_2_ according to the following equations:

4 $$ mqe^{\left( \sigma \right)} < \tau_{1} qe_{\xi }^{{\left( \sigma \right)_{{{\mathrm{parent}}}} }} $$

5 $$ qe_{e}^{\left( \sigma \right)} < \tau_{2} qe_{1}^{\left( 0 \right)} $$

where *mqe* represents mean quantisation error, *qe* is quantisation error, and ξ is the index of the neuron of the parent map σ. This resulted in a GHSORM model that promoted architectural depth over the size of individual SOMs in the model. The Pre-Processor GHSORM, wherein all SOMs in the model share a DA, was used because this resulted in maximal Normalised Mutual Information between clusters. GHSORM parameters used in this study can be found in Table 2. Pseudocode describing the GHSORM algorithm can be found as Supplementary Text 1.

**Table 2:** Optimal treatment arms and maximum %pCR for I-SPY2 clinical trial patients grouped according to GEC, RPS-5, and Receptor Subtype

Subtyping schema	Subtype	Optimal_Arm	n_patients	pCR_count	pCR_max_STi	prev_STi	prev_STi x pCR_max_STi
GEC	GEC1	Paclitaxel + MK-2206 + Trastuzumab	1	1	1	0.1412602	0.1412602
	GEC2	T-DM1 + Pertuzumab	10	6	0.6	0.1219512	0.07317072
	GEC3	Paclitaxel + Pertuzumab + Trastuzumab	2	1	0.5	0.1006098	0.0503049
	GEC4	Paclitaxel + Pertuzumab + Trastuzumab	3	3	1	0.2388211	0.2388211
	GEC5	Paclitaxel + Pembrolizumab	9	9	1	0.1554878	0.1554878
	GEC6	Paclitaxel + AMG 386 + Trastuzumab	5	4	0.8	0.1107724	0.08861792
	GEC7	Paclitaxel + MK-2206 + Trastuzumab	6	5	0.8333333	0.1310976	0.109247996
RPS-5	HER2+/BP-HER2_or_Basal	Paclitaxel + Pertuzumab + Trastuzumab	32	25	0.78125	0.18800813	0.146881352
	HER2+/BP-Luminal	Paclitaxel + MK-2206 + Trastuzumab	5	3	0.6	0.06097561	0.036585366
	HER2-/Immune +	Paclitaxel + Pembrolizumab	34	27	0.7941176	0.38109756	0.30263628
	HER2-/Immune-/DRD.v3 +	Paclitaxel + ABT 888 + Carboplatin	10	6	0.6	0.07926829	0.047560974
	HER2-/Immune-/DRD.v3-	Paclitaxel + MK-2206	20	3	0.15	0.28760163	0.043140245
Receptor Subtype	HR + HER2 +	T-DM1 + Pertuzumab	35	18	0.5142857	0.15853659	0.081533101
	HR + HER2-	Paclitaxel + Pembrolizumab	40	12	0.3	0.38313008	0.114939024
	HR-HER2 +	Paclitaxel + Pertuzumab + Trastuzumab	15	12	0.8	0.09044715	0.07235772
	TN	Paclitaxel + Pembrolizumab	29	19	0.6551724	0.36788618	0.241028871

The aweSOM [49] and Kohonen [50] R-packages were used to visualise GHSORM output. Hierarchical superclass clustering of the SOM codebook vectors was performed to determine which SOM neurons belonged in a GEC. DynamicTreeCut [51] was used to group SOM nodes into GECs.

### Recursive Feature Elimination with Strikeout (RFES)

To identify the minimum set of glycogenes that can classify the GECs with high accuracy, a Recursive Feature Elimination with Strikeout (RFES) feature discovery wrapper was designed around the Random Forest algorithm. The process commenced with the complete set of 341 glycogenes. In every iteration, unimportant glycogenes were eliminated until model accuracy decreased *s* times. This *strikeout* parameter, inspired by the tuning step in the RandomForestSRC framework [52], introduces flexibility into the model by providing a tolerance in the accuracy search such that a decrease in improvement is allowed *s* times before termination. This allows the accuracy to fluctuate prior to locating the ideal gene set (Supplementary Figure S7).

RFES was run using the RandomForest algorithm [53] in the caret [54, 55] package. The model was trained on 80% of the data using a 5-fold cross validation approach and tested on the remaining 20%. Values of *ntree* included 300, 500, and 1000, and an *mtry* from 1 to 40 was used. Computational time was measured with the tictoc R-package [56].

To avoid a decline in Random Forest classification accuracy due to class imbalanced data, majority classes (n > 112) were randomly undersampled and minority classes (n < 112) were artificially inflated with RACOG [57]. RFES with this sampling approach was benchmarked against three commonly used feature discovery algorithms: Recursive Feature Elimination (RFE), Simulated Annealing (SA), and Genetic Algorithms (GA) [55]. RFES performed the best, although it ran significantly slower than the other algorithms. This is in part because the RFES algorithm repeats feature discovery three times to avoid inherent algorithmic randomness and produce stable feature sets (Supplementary Table S4).

The RFES algorithm proceeds as follows:

*s* and Accuracy_*max*_ are initialised to 0.

The feature importance for each gene across all GECs in data_train_ is obtained using the varImp function from the caret package [54, 55], and the maximum variable importance, as Mean Decrease in Accuracy (MDA) for each gene (*j*) across the GECs *(m)* is recorded.

6 $$ {\mathrm{MDA}}_{j,max} = \max \left( {{\mathrm{MDA}}_{j,1} ,{\mathrm{MDA}}_{j,2} , \ldots ,{\mathrm{MDA}}_{j,m} } \right) $$

The first quantile (*Q*_*1*_) is found from the maximum VI across all features, where *p* is the total number of features.

7 $$ Q_{1 } = {\mathrm{Quantile}}_{0.25} \left( {{\mathrm{MDA}}_{1,max} , {\mathrm{MDA}}_{2,max} , \ldots ,{\mathrm{MDA}}_{p,max} } \right) $$

The features (*F*) are retained if they have a maximum MDA greater than the first quantile.

8 $$ F = {(}j {|} {\mathrm{MDA}}_{j,max} > Q_{1} ) $$

Using this feature set *F,* the model is run again and the accuracy of the model on data_train_ and data_test_ are recorded as follows:

9 $$ {\mathrm{Accuracy}} = \frac{{{\mathrm{True}}\; {\mathrm{Positives}} + {\mathrm{True}}\; {\mathrm{Negatives}}}}{{{\mathrm{True}}\; {\mathrm{Positives}} + {\mathrm{True}}\; {\mathrm{Negatives}} + {\mathrm{False}}\;{\text{ Positives}} + {\mathrm{False}}\;{\text{ Negatives}}}} $$

If Accuracy_*test*_ (model accuracy on data_test_) is greater than Accuracy_*max*_ then Accuracy_*max*_ = Accuracy_*test*_, else *s* is incremented by 1. This is repeated until *s* > *strikeout*.

Repeat the procedure *rpt* times and record the most accurate model for each repeat.

An important note is that data_test_ does not include artificial samples from the RACOG, such that the accuracy of the model is only evaluated on real samples.

### Immune cell deconvolution

The online version of Cibersort [58] was used in relative mode for immune cell deconvolution, utilising the LM22 signature matrix file and the TMM-normalised counts as input. Batch correction was enabled, quantile normalisation was disabled, and 1000 permutations were used for significance analysis. Only the 773 samples with a p-value < 0.05 were utilised in the analysis.

ssGSEA [59] was employed with the GSVA package [60] to calculate glycosylation pathway scores for the investigation of correlations between glycosylation pathways and estimated relative immune cell abundance in each GEC. Only cells with significantly different abundances according to the linear model (p-adjusted < 0.05 and correlation coefficients > 0.25) were included. To determine which correlations to include in the figure, the mean of correlations for cell – glycopath combinations in each GEC were calculated, and only those combinations with a mean_correlation_ > 0.25 were included. This was done focus on the pathways that varied most between GECs; however, Supplementary Table S3 contains the correlations for all cell – glycopath combinations.

### Gene regulatory network

Arboreto [61] was used for the construction of a gene regulatory network, using the vst-normalised count matrix as input. The 60 glycogenes identified by RFES as important (Supplementary Table S1) were used as the target genes, and the expression of the remainder of the genes with a SD > 0.5 was predicted. Arboreto produces a list of weights representing the importance of a target gene in predicting the expression of a non-target gene. High weights are potentially indicative of a functional relationship between the two genes.

For each GEC, important (RFES importance score > 50) and truly upregulated glycogenes – as compared to normal tissue and to all other clusters (p-adj < 0.05; LFC > 0) – were extracted from the Arboreto output. The non-target genes that these glycogenes could predict (Arboreto score > 5) were subjected to over-representation analysis with ClusterProfiler [62, 63] using all the input genes as the background. Pathways with a p-value < 0.05 were considered significant and the combined functional analysis across all GECs was used as input to Cytoscape [64].

### Predicting percentage pCR

To determine the best treatment in each GEC and the overall pCR rate had patients been grouped according to GEC, we followed the same method stipulated in [9]:

The prevalence of each GEC was calculated as:

10 $$ {\mathrm{prev}}_{STi} = \frac{{{\mathrm{Number}}\;{\text{ patients}} \;{\mathrm{in}}\;{\text{ GEC}}}}{{{\mathrm{Number}}\;{\text{ of}}\;{\text{ GECs}}}} $$

The highest pCR rates observed per GEC was collected, and denoted pCR*_max_STi.*

The maximum pCR per GEC was calculated as:

11 $$ {\mathrm{pCR}}_{{max_{total } }} = \mathop \sum \limits_{i = 1}^{n} {\mathrm{prev}}_{STi} \times {\text{ pCR}}\_{\mathrm{max}}\_{\mathrm{STi}} $$

### Other software packages

Survival was analysed using and the survival [65], GGally [66], and survminer [67] R packages. Heatmaps were drawn using ComplexHeatmap [68]. Dendsort [69] and circlize [70] were used for hierarchical clustering of heatmap rows and columns, and generation of colour vectors respectively. Rstatix was used for statistical testing [71]. The pROC package was used to construct Receiver Operating Characteristic curves [72]. Functional analysis and pathway enrichment was performed using the Gene Ontology database [73, 74], except in the case of glycosylation pathways, which were obtained from GlycoEnzOnto [44], and glycogene function was researched with GlycoEnzDB [75]. The Reactome [76, 77] and Genecards [78, 79] databases were frequently used to look up gene function.

### Multiple testing correction

All p-values were corrected with the Benjamini–Hochberg procedure, and the significance threshold used for statistical testing is adjusted p-value < 0.05 unless otherwise stated.

## Supplementary Information

Below is the link to the electronic supplementary material.

Supplementary File 1.

## Data Availability

The results shown here are based upon data generated by the TCGA Research Network: https://www.cancer.gov/tcga and the I-SPY2 clinical trial [9] (Gene Expression Omnibus Accession code GSE194040). All R code used in this study will be made available upon request.

## Abbreviations

DBI: Davies-Bouldin Index
GEC: Glycogene Expression Cluster
GHSORM: Growing Hierarchical Self-Organising Representation Map
NMI: Normalised Mutual Information
RFES: Recursive Feature Elimination with Strikeout
