# Supplementary material for: Unsupervised machine learning identifies early stage breast cancer subclasses from aberrant glycogene expression and informs personalised drug treatment
Source: Discov Chem. 2026 Nov 14;3(1):630. doi: 10.1007/s44371-026-01070-0 (PMC13769728; doi:10.1007/s44371-026-01070-0)
Supplement: Supplementary file 1 [file 44371_2026_1070_MOESM1_ESM.pdf]

Supplementary Text 1: Pseudocode for GHSORM algorithm

```
Algorithm: Growing Hierarchical Self-Organising Representation Map (GHSORM)

INPUT:
  X_raw ∈ ℝ^(n×d) - Raw input dataset (n samples, d features)
  η_init - Initial learning rate
  σ_init - Initial neighborhood radius
  min_nodes - Minimum nodes per SOM layer
  max_nodes - Maximum nodes per SOM layer
  growth_threshold - Threshold for node splitting

OUTPUT:
  Hierarchical cluster structure with multi-level representations

================================================================================
PHASE 1: DENOISING AUTOENCODER PREPROCESSING
================================================================================

FUNCTION TrainDenoisingAutoencoder(X_raw, noise_level, epochs):
  // Initialize DA weights
  W_enc ← RandomInitialize(weights_encoder)
  W_dec ← RandomInitialize(weights_decoder)

  FOR epoch = 1 TO epochs:
    FOR each mini-batch B in X_raw:
      // Corruption step - add noise to input
      X_corrupted ← AddNoise(B, noise_level)
```

```
        // Forward pass through encoder
        H_encoded ← Encoder(X_corrupted, W_enc)

        // Forward pass through decoder (reconstruction)
        X_reconstructed ← Decoder(H_encoded, W_dec)

        // Compute reconstruction loss
        Loss ← ReconstructionLoss(B, X_reconstructed)

        // Backpropagation to update weights
        UpdateWeights(W_enc, W_dec, Loss, learning_rate)

    RETURN W_enc, W_dec

FUNCTION ExtractRepresentations(X_raw, W_enc):
    // Use trained encoder to extract robust feature representations
    X_processed ← Encoder(X_raw, W_enc)
    RETURN X_processed
```

```
================================================================================
PHASE 2: GROWING HIERARCHICAL SOM CLUSTERING
================================================================================

CLASS SOMNode:
  Attributes:
    weight_vector ∈ ℝ^m - Node weights (m = encoded dimension)
    children[] - List of child nodes (for hierarchy)
    data_points[] - Samples assigned to this node
    activation_count - Number of activations

  METHODS:
    UpdateWeights(input, neighborhood_strength):
      // Standard SOM weight update with neighborhood function
      FOR each neighbor n in GetNeighbors(self, σ):
        strength ← NeighborhoodFunction(distance_to_cmu, σ)
        n.weight_vector ← n.weight_vector + η × strength × (input - n.weight_vector)

FUNCTION InitializeRootSOM(X_processed, min_nodes):
  // Create initial SOM layer with minimum nodes
  root_layer ← New SOMLayer()

  FOR i = 1 TO min_nodes:
    node ← New SOMNode(weight_dimension = X_processed.shape[1])
    node.weight_vector ← RandomSampleFrom(X_processed)
    root_layer.AddNode(node)

  RETURN root_layer
```

```
FUNCTION FindBestMatchingUnit(layer, input):
    // Find node with minimum distance to input
    min_distance ← ∞
    bmu ← NULL

    FOR each node in layer.nodes:
        distance ← EuclideanDistance(input, node.weight_vector)
        IF distance < min_distance:
            min_distance ← distance
            bmu ← node

    RETURN bmu

FUNCTION UpdateNodeAndNeighbors(layer, input, bmu, η, σ):
    // Update BMU and its neighbors using Gaussian neighborhood function
    FOR each node in layer.nodes:
        distance ← EuclideanDistance(bmu.position, node.position)
        neighborhood_strength ← exp(-distance² / (2 × σ²))

        IF neighborhood_strength > threshold:
            node.UpdateWeights(input, η × neighborhood_strength)

FUNCTION CheckGrowthCondition(layer):
    // Determine if layer needs to grow based on quantization error
    total_error ← 0

    FOR each data_point in layer.data_points:
        bmu ← FindBestMatchingUnit(layer, data_point)
        error ← EuclideanDistance(data_point, bmu.weight_vector)
```

```
        total_error ← total_error + error²

    avg_error ← total_error / length(layer.data_points)

    RETURN avg_error > growth_threshold

FUNCTION SplitNode(layer, node_to_split):
    // Create new nodes by splitting an existing node
    new_node_1 ← New SOMNode()
    new_node_2 ← New SOMNode()

    // Initialize weights with small perturbation from parent
    new_node_1.weight_vector ← node_to_split.weight_vector + ε₁
    new_node_2.weight_vector ← node_to_split.weight_vector + ε₂

    layer.AddNodes(new_node_1, new_node_2)

    // Reassign data points to nearest of the three nodes
    FOR each point in node_to_split.data_points:
        closest ← FindClosestNode([node_to_split, new_node_1, new_node_2], point)
        closest.AddDataPoint(point)

FUNCTION GrowLayer(layer, X_processed_subset):
    // Expand layer by adding nodes until convergence or max reached
    WHILE length(layer.nodes) < max_nodes AND CheckGrowthCondition(layer):

        // Find node with highest quantization error (candidate for splitting)
        worst_node ← FindHighestErrorNode(layer)
```

```
    IF worst_node is not NULL:
        SplitNode(layer, worst_node)

        // Reorganize data points in affected region
        ReassignDataPoints(layer, X_processed_subset)

FUNCTION CreateHierarchicalLayer(parent_layer, X_processed):
    // Create child layer for sub-clustering of parent nodes

    child_layer ← New SOMLayer()

    FOR each node in parent_layer.nodes:
        IF length(node.data_points) > min_samples_for_subclustering:
            // Extract subset belonging to this parent node
            subset ← node.data_points

            // Initialize small SOM for this subset
            sub_som ← InitializeRootSOM(subset, min_nodes_per_child)

            // Grow the sub-SOM
            GrowLayer(sub_som, subset)

            // Link child layer to parent node
            node.children.Add(sub_som)

    RETURN child_layer
```

```
================================================================================
PHASE 3: GHSORM MAIN EXECUTION
================================================================================

FUNCTION GHSORM(X_raw, config):
    // Configuration parameters
    noise_level ← config.noise_level
    da_epochs ← config.da_epochs
    min_nodes ← config.min_nodes
    max_nodes ← config.max_nodes
    growth_threshold ← config.growth_threshold
    hierarchy_depth ← config.hierarchy_depth

    // STEP 1: Preprocess with Denoising Autoencoder
    PRINT "Training Denoising Autoencoder..."
    W_enc, W_dec ← TrainDenoisingAutoencoder(X_raw, noise_level, da_epochs)

    PRINT "Extracting robust feature representations..."
    X_processed ← ExtractRepresentations(X_raw, W_enc)

    // STEP 2: Initialize root SOM layer
    PRINT "Initializing hierarchical SOM structure..."
    current_layer ← InitializeRootSOM(X_processed, min_nodes)

    // STEP 3: Train and grow root layer
    PRINT "Growing root SOM layer..."
    GrowLayer(current_layer, X_processed)

    // STEP 4: Create hierarchical sub-layers recursively
```

```
    FOR depth = 1 TO hierarchy_depth - 1:
        PRINT "Creating hierarchical level", depth + 1

        new_layers ← []

        FOR each node in current_layer.nodes:
            IF length(node.data_points) > min_samples_for_subclustering:
                // Create sub-layer for this cluster
                sub_layer ← InitializeRootSOM(node.data_points, min_nodes)
                GrowLayer(sub_layer, node.data_points)

                // Store hierarchical relationship
                node.children.Add(sub_layer)
                new_layers.Add(sub_layer)

        current_layer ← CombineLayers(new_layers)

    // STEP 5: Return complete hierarchical structure
    RETURN current_layer

================================================================================
SUPPORTING FUNCTIONS FOR VISUALIZATION AND ANALYSIS
================================================================================

FUNCTION NodeActivationMaximisation(layer, target_node):
    // Generate visual representation of what activates a specific node
    // (As mentioned in the paper for digit visualization)

    Initialize random input I ∈ ℝ^m
```

```
    FOR iteration = 1 TO max_iterations:
        Compute gradient ∇I Loss(target_node.activation(I))
        Update I ← I + learning_rate × ∇I

        Apply constraints to keep I within valid range

    RETURN I (visual representation of node's receptive field)

FUNCTION ExtractClusterHierarchy(root_layer):
    // Traverse and extract hierarchical cluster structure
    hierarchy ← New TreeStructure()

    Function Traverse(node, parent_cluster):
        cluster_id ← GenerateUniqueID()

        FOR each child_som in node.children:
            FOR each child_node in child_som.nodes:
                sub_cluster_id ← GenerateUniqueID()
                AddToHierarchy(hierarchy, cluster_id, sub_cluster_id)
                Traverse(child_node, sub_cluster_id)

    Traverse(root_layer, root_cluster)
    RETURN hierarchy

================================================================================
END OF GHSORM ALGORITHM
================================================================================
```

Supplementary Figures

A

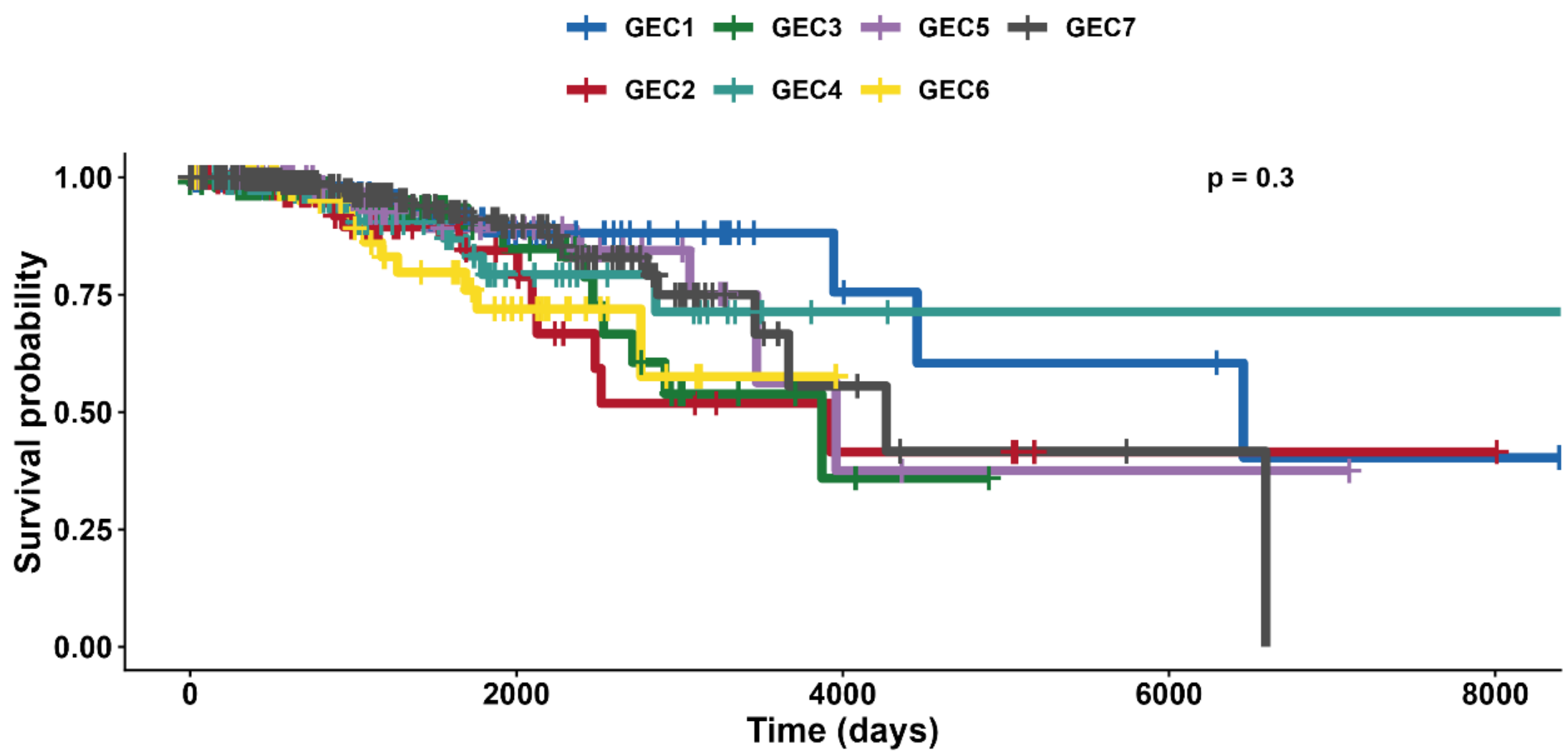

B

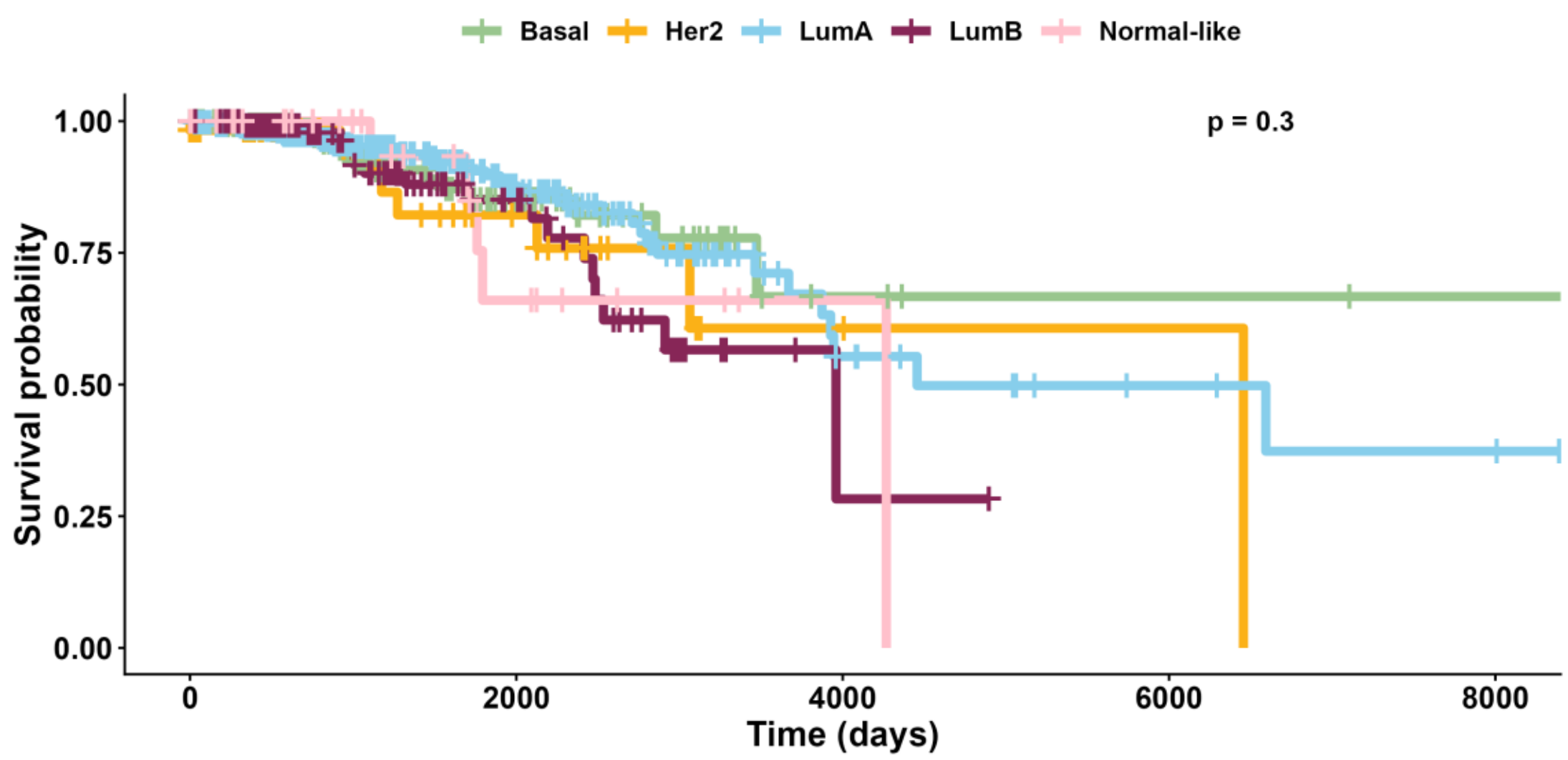

Supplementary Figure S1: Kaplan-Meier survival curves for **(A)** GECs and **(B)** PAM50 subtypes. P-values indicated on the plots were calculated with the Log-rank test.

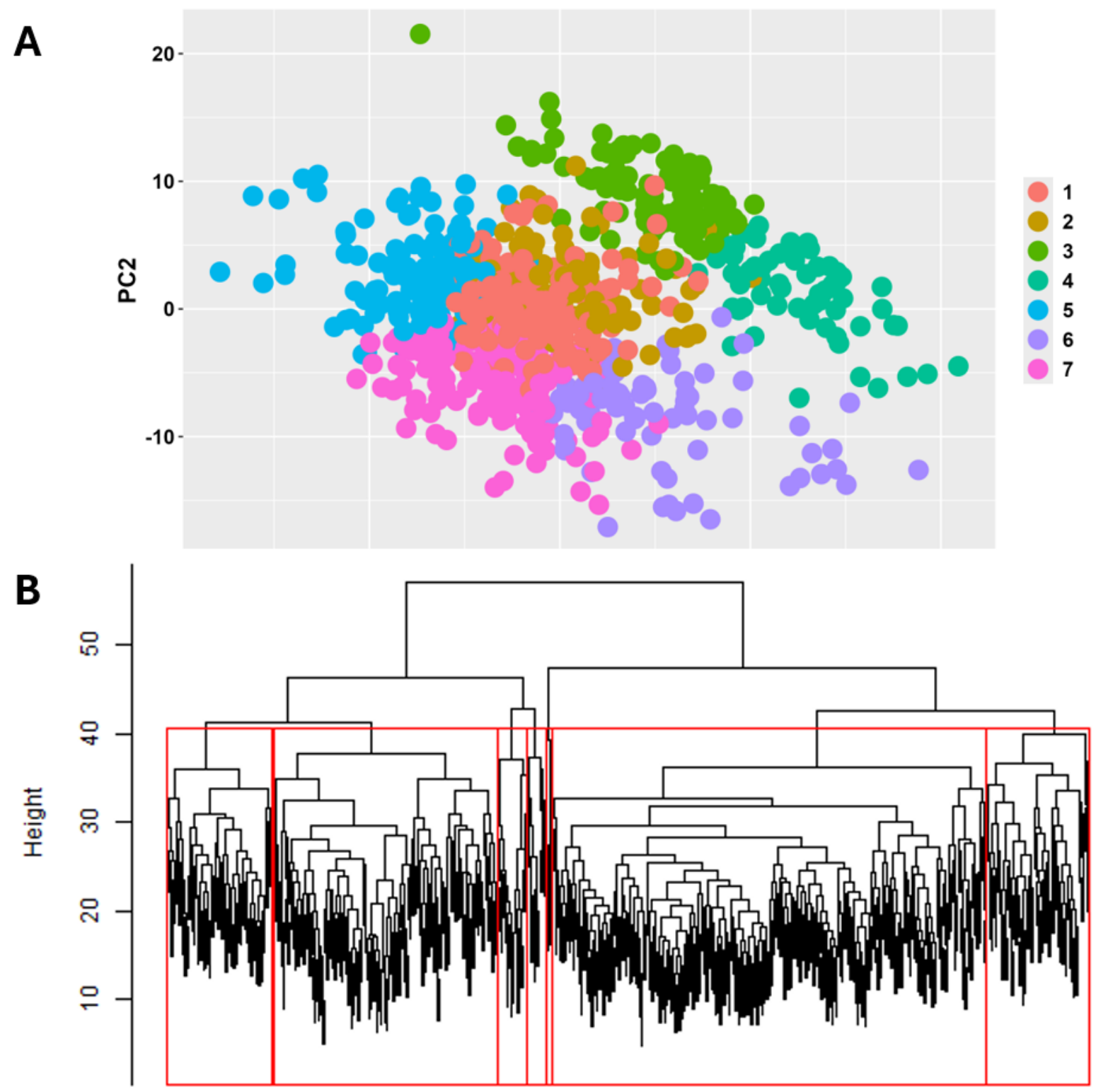

Supplementary Figure S2: Clustering of the TCGA samples (n = 785) using **(A)** K-means clustering using 7 centers (DBI = 3.26) and **(B)** Hierarchical clustering with Euclidean distance and complete linkage (DBI = 3.5).

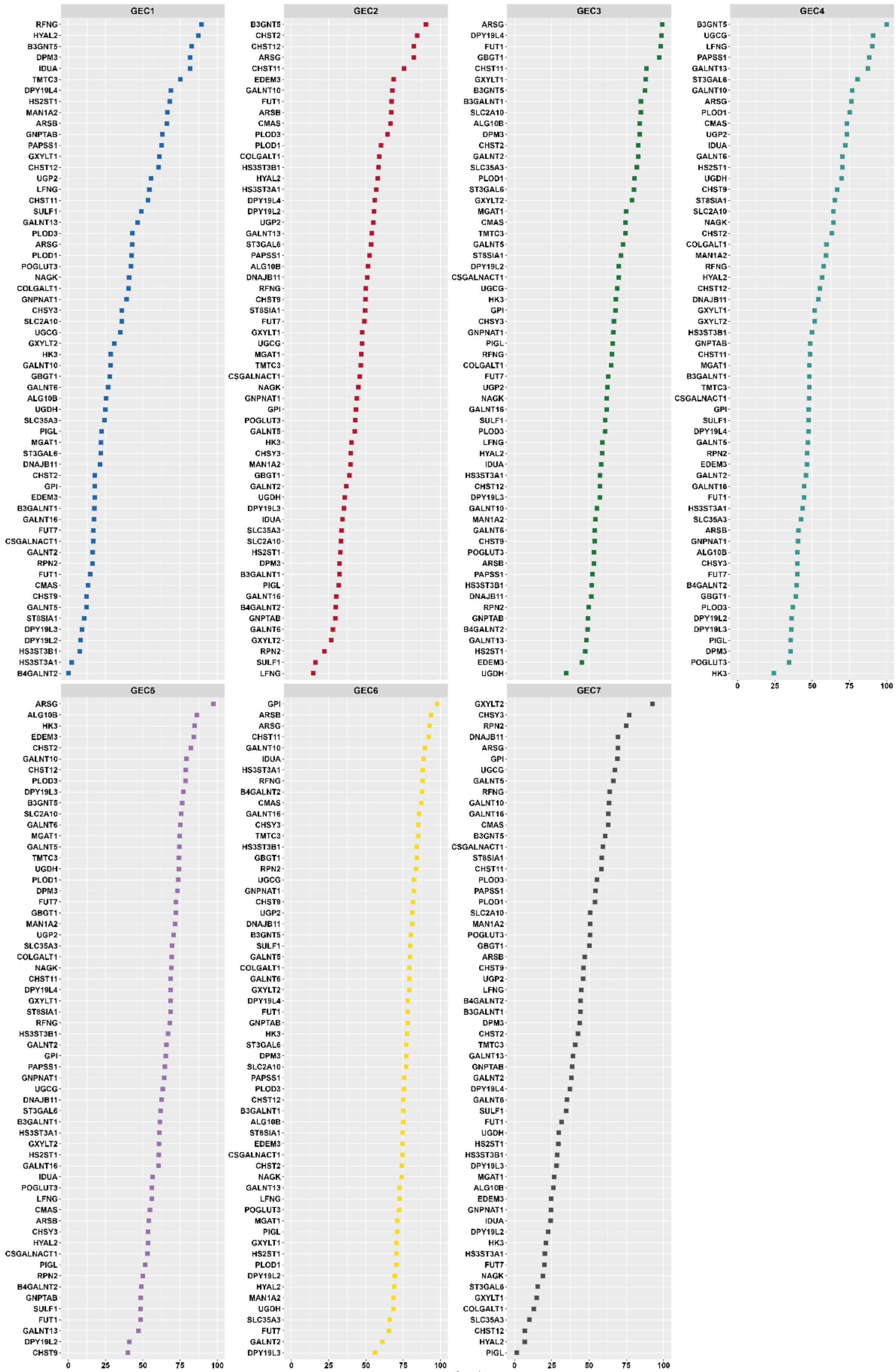

Supplementary Figure S3: RFES-derived feature importance for the 60 chosen glycogenes in each GEC.

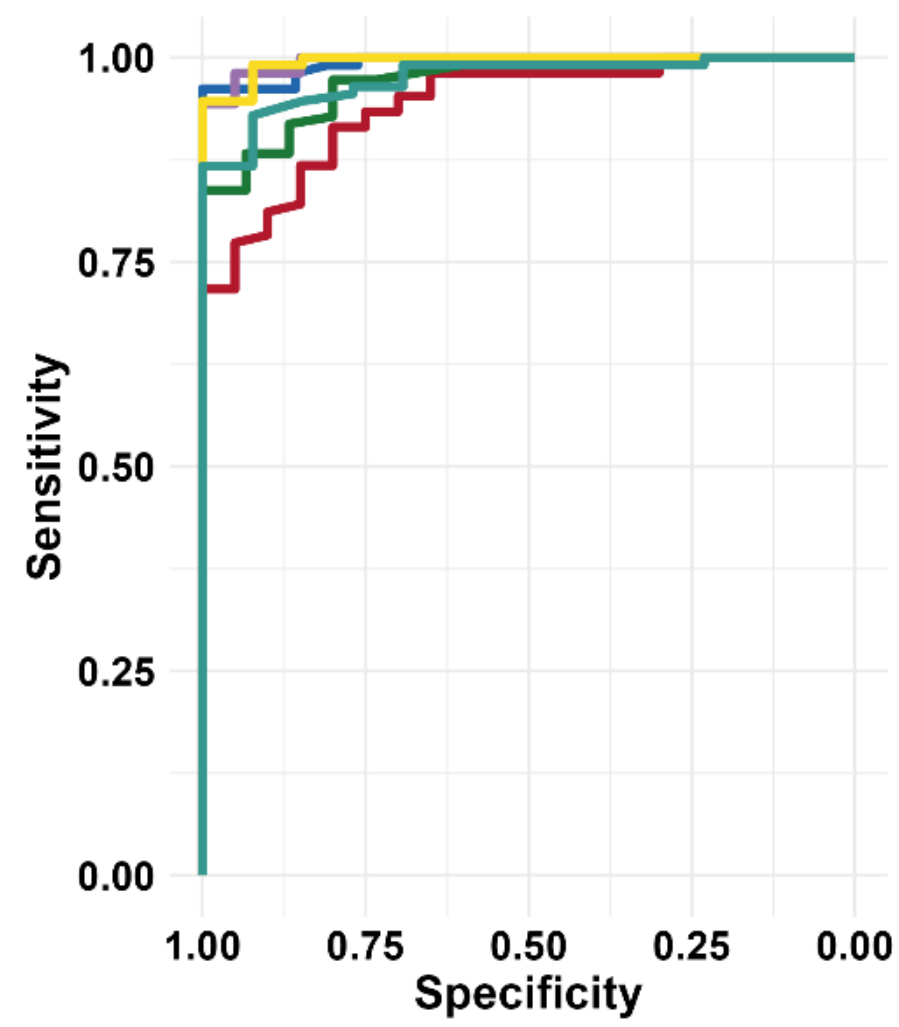

Supplementary Figure S4: Receiver Operating Characteristics curve showing performance of the Random Forest classifier for each cluster on the independent testing set.

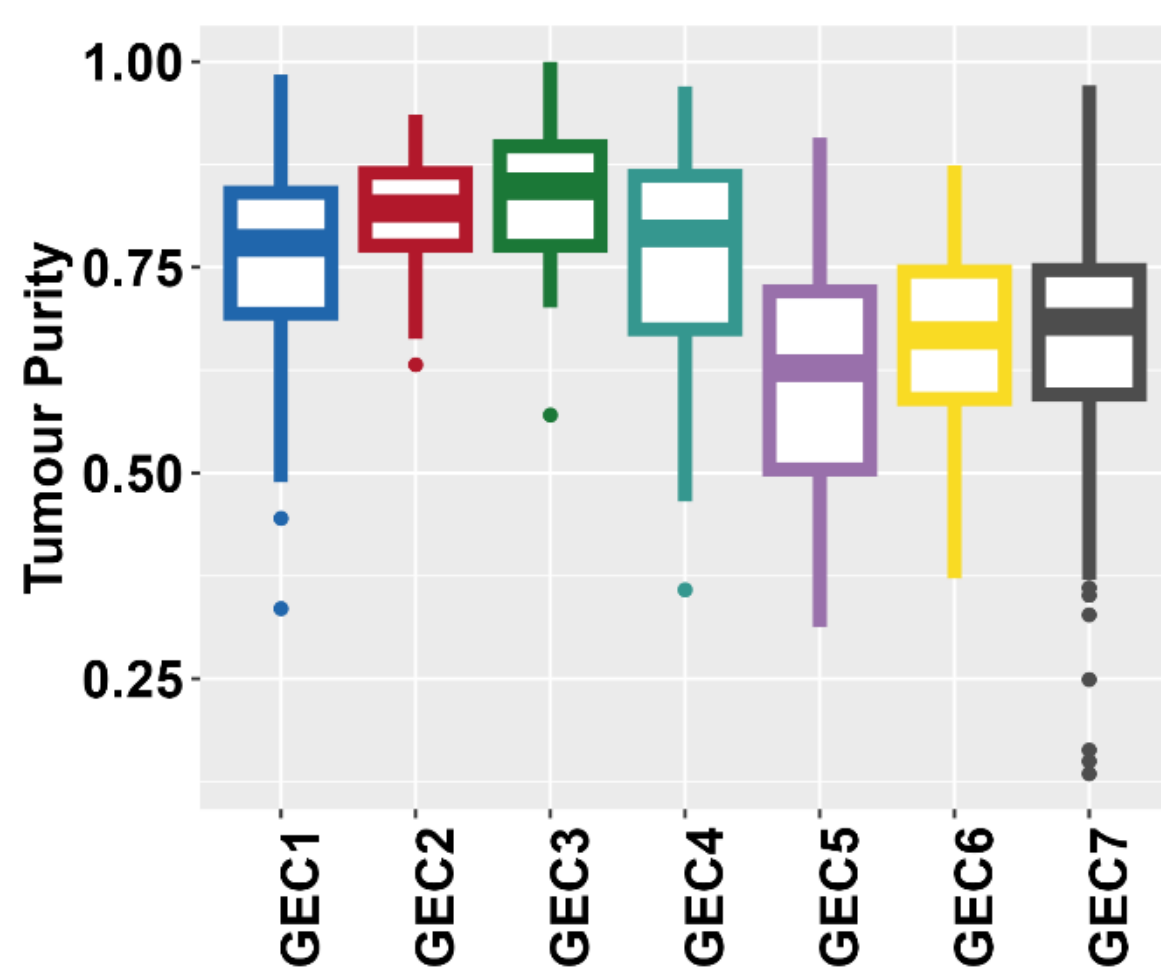

Supplementary Figure S5: Tumour purity across the GECs. Kruskal-Wallis p-value <2 x10^{-16}

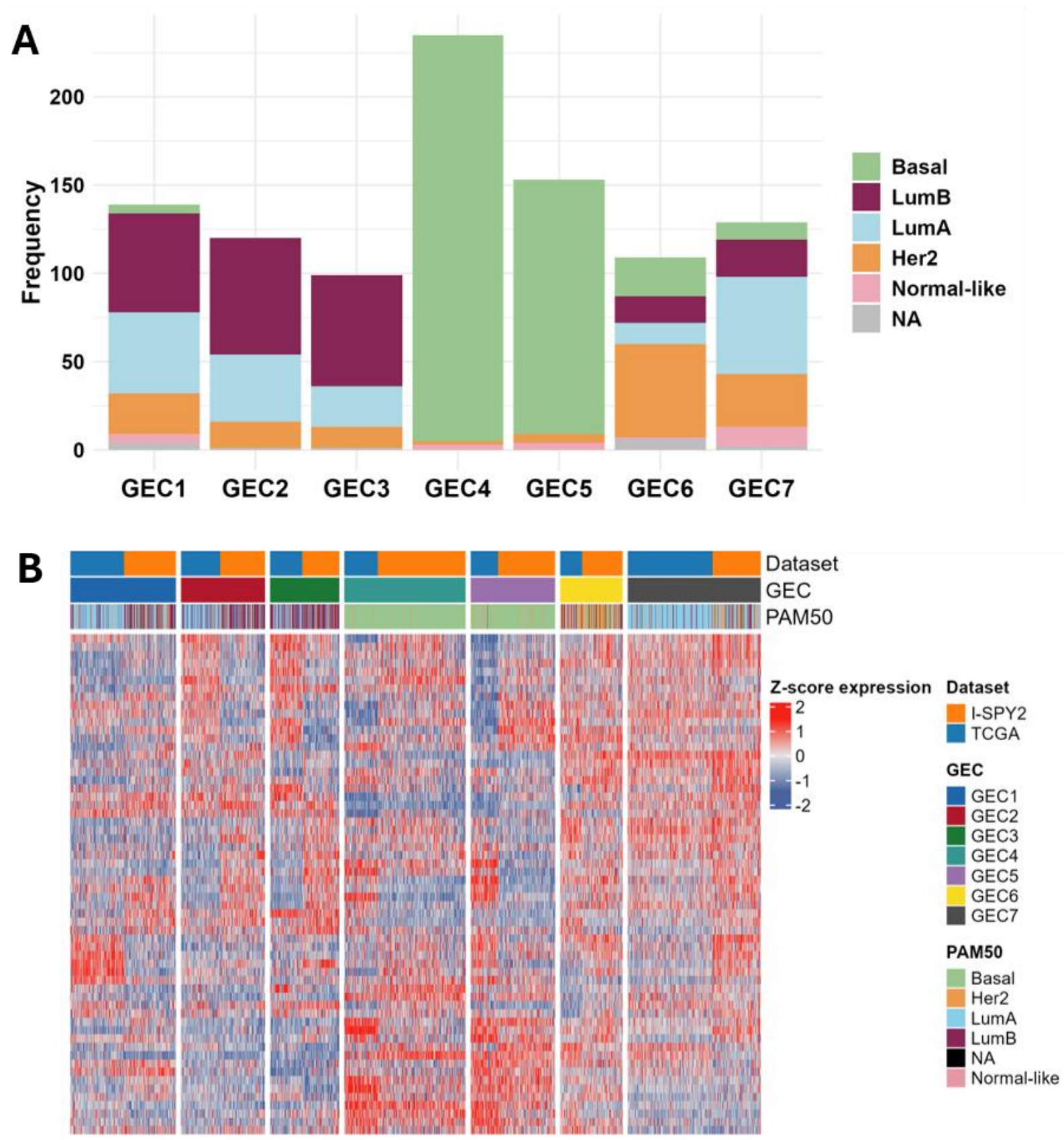

Supplementary Figure S6: GECs in the I-SPY2 clinical trial as predicted by the trained Random Forest classifier using the 60-glycogene panel. **(A)** Distribution of PAM50 subtypes per GEC. **(B)** Heatmap showing expression of the 60 glycogenes in each GEC for both the I-SPY2 and TCGA datasets. Dataset is denoted as orange (I-SPY2) and blue (TCGA) bars in the top annotation of the heatmap.

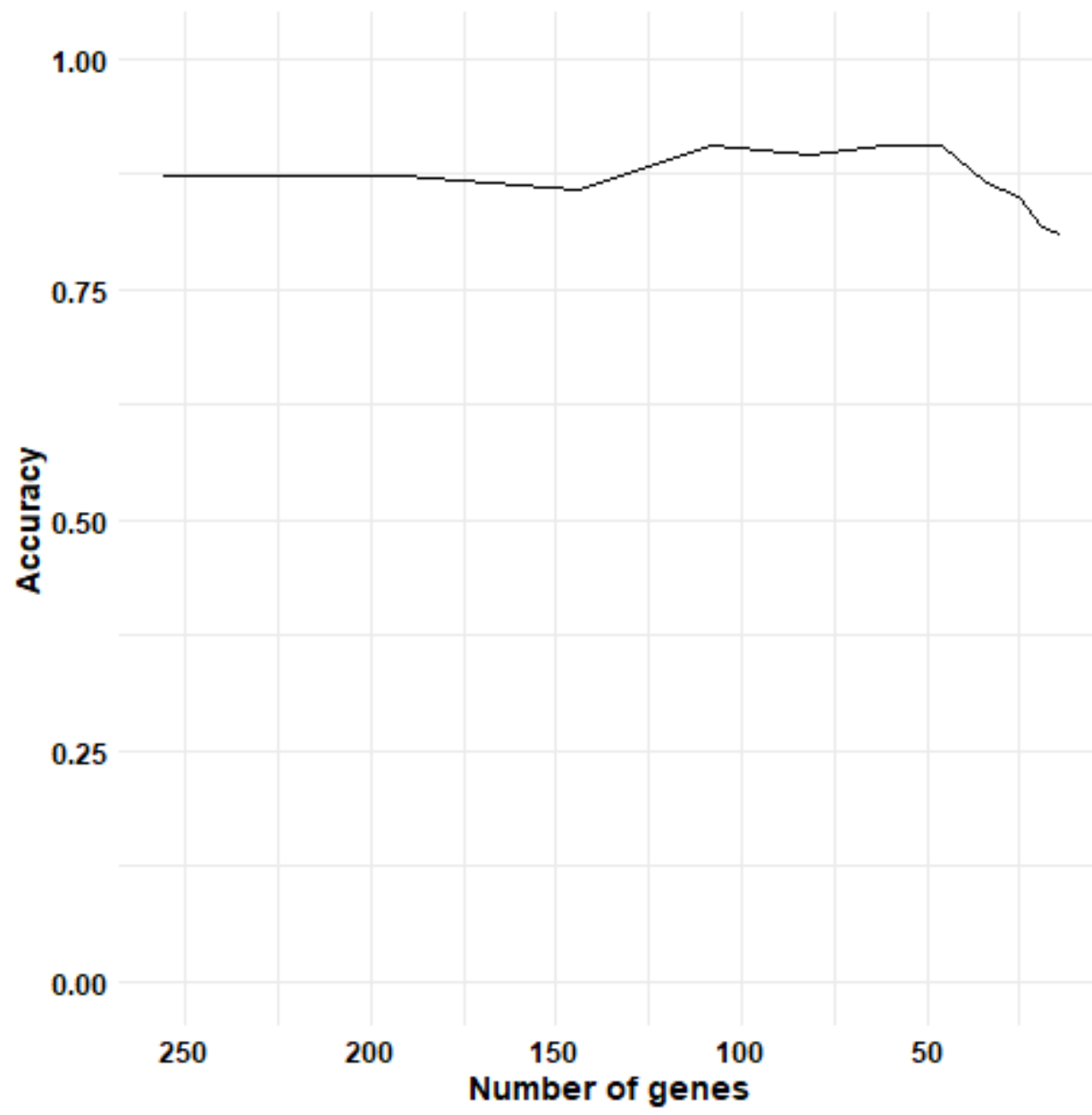

Supplementary Figure S7: Accuracy fluctuation in the RFES model prior to finding the ideal gene set.
